# Data fusion of body-worn accelerometers and heart rate to predict VO_2max_ during submaximal running

**DOI:** 10.1371/journal.pone.0199509

**Published:** 2018-06-29

**Authors:** Arne De Brabandere, Tim Op De Beéck, Kurt H. Schütte, Wannes Meert, Benedicte Vanwanseele, Jesse Davis

**Affiliations:** 1 Department of Computer Science, KU Leuven, Leuven, Belgium; 2 Department of Movement Sciences, KU Leuven, Leuven, Belgium; 3 Department of Sport Sciences, Stellenbosch University, Stellenbosch, South Africa; University of Colorado Boulder, UNITED STATES

## Abstract

Maximal oxygen uptake (VO_2max_) is often used to assess an individual’s cardiorespiratory fitness. However, measuring this variable requires an athlete to perform a maximal exercise test which may be impractical, since this test requires trained staff and specialized equipment, and may be hard to incorporate regularly into training programs. The aim of this study is to develop a new model for predicting VO_2max_ by exploiting its relationship to heart rate and accelerometer features extracted during submaximal running. To do so, we analyzed data collected from 31 recreational runners (15 men and 16 women) aged 19-26 years who performed a maximal incremental test on a treadmill. During this test, the subjects’ heart rate and acceleration at three locations (the upper back, the lower back and the tibia) were continuously measured. We extracted a wide variety of features from the measurements of the warm-up and the first three stages of the test and employed a data-driven approach to select the most relevant ones. Furthermore, we evaluated the utility of combining different types of features. Empirically, we found that combining heart rate and accelerometer features resulted in the best model with a mean absolute error of 2.33 ml ⋅ kg^−1^ ⋅ min^−1^ and a mean absolute percentage error of 4.92%. The model includes four features: gender, body mass, the inverse of the average heart rate and the inverse of the variance of the total tibia acceleration during the warm-up stage of the treadmill test. Our model provides a practical tool for recreational runners in the same age range to estimate their VO_2max_ from submaximal running on a treadmill. It requires two body-worn sensors: a heart rate monitor and an accelerometer positioned on the tibia.

## Introduction

In endurance sports such as distance running and cycling, there is a large interest among coaches and sports scientists to monitor the cardiorespiratory fitness of athletes for both inter- and intra-athlete comparison. This is often measured in terms of the maximal oxygen uptake (VO_2max_), which is defined as the maximal rate at which an individual can consume oxygen during exercise. VO_2max_ is one of the primary determinants of endurance performance [1], alongside the fractional utilization of VO_2max_ (lactate threshold), and economy of movement [2].

Typically, VO_2max_ is measured by performing a maximal incremental running test on a treadmill. However, VO_2max_ testing is often too expensive for non-elite athletes, since maximal exercise tests must be administered by trained staff in a lab set-up with specialized equipment. According to the ACSM guidelines on exercise testing [3], the staff should be capable of recognizing contraindications to performing maximal exercise tests and to interpret an electrocardiogram (ECG) as the participants exercise until volitional exhaustion. Moreover, for athletes who follow a training program, it may be hard to incorporate maximal tests regularly into their training plan, as these may interfere with the planned training sessions.

These limitations have motivated the development of models that can predict VO_2max_ from submaximal exercise. An extensive overview by Abut et al. [4] compares various maximal, submaximal, and non-exercise models. We focus on the latter two since we are predicting VO_2max_ from submaximal exercise. Typically, the models are constructed by viewing this as a regression problem. Thus the two key design choices are selecting the model class, and defining the relevant predictor variables. In terms of model class, most approaches use linear regression but some studies have considered support vector regression and artificial neural networks. The predictor variables (“features”) used in existing models fall in two categories: non-exercise features and features collected during submaximal exercise.

In Abut et al.’s overview, all models included **non-exercise features** such as gender, age, body mass, height, and BMI. Some studies also considered features based on questionnaire responses such as the perceived functional ability [5–8] (i.e., a person’s self-reported ability to walk, jog or run at a comfortable pace for 1 mile (1.609 km) and for 3 miles (4.828 km)), and physical activity rating [9] (i.e., a person’s self-rated physical activity level during the past 6 months).

Several studies have also included **features collected during submaximal exercise** [10–15] by measuring the average heart rate during walking or running, or the heart rate at the end of exercising for a set time or distance. Furthermore, these are often augmented with features such as the time needed to cover a set distance, the distance covered in a fixed time period [11–13], or features extracted from accelerometer signals [14, 15]. Weyand et al. [14] considered the average heart rate (HR) and the inverse of the foot-ground contact time ($t_{c}^{-1}$) as measured by a specifically designed, non-commercial, foot-based accelerometer during running. Tönis et al. [15] considered heart rate and the “level of activity” during walking at two different velocities. The level of activity was defined as the sum of the integrals of the absolute value of the acceleration for the three accelerometer axes. Other studies have used the relation between heart rate and features derived from accelerometer data by monitoring subjects in free-living conditions. The accelerometer features in these studies included total acceleration [16], accelerometer counts [17, 18], step counts [19, 20], and walking speed derived from the acceleration signals [21].

The existing approaches for predicting VO_2max_ have several limitations. Many of the models [5–8] rely on subjective features collected from questionnaires, and an individual’s poor or misleading answers may unduly affect the results. Including features derived from heart rate and accelerometer sensors overcomes this drawback, as these sensors are considered to be objective methods for monitoring physical activity [22, 23]. They have been used in previous studies based on submaximal running for several minutes [14], walking for a fixed duration [15], and free-living conditions where participants wore sensors throughout the day without adhering to a specific protocol [16–21]. Besides using objective measurements, these methods also allow estimating VO_2max_ from daily activities. While most of these studies rely on data measured during a full day, or even multiple days, those by Weyand et al. [14] and Tönis et al. [15] only need several minutes of exercise. We argue that a short protocol offers the advantage of making it easier to incorporate VO_2max_ estimation into exercise routines, since it only requires an individual to wear the sensors for a short amount of time instead of a full day. However, both of these studies have their limitations as well. Tönis et al. did not verify their model’s predictions using subjects’ true VO_2max_. Instead, they checked how well their model’s predictions correlated with a subject’s estimated VO_2max_ as determined by a submaximal walking test [24]. Therefore, it is unclear how accurate their model is in practice. Weyand et al.’s model relies on using a specialized foot-based accelerometer to measure foot-ground contact time. This requires buying a specialized sensor with a high sampling rate, which recreational athletes may not want to do. Both Weyand et al. and Tönis et al. only included one or two hand-selected features in their model. Particularly when confronted with multi-sensor data, it is difficult for a domain expert to hand select all the relevant features that should be included in a model.

To address these limitations, this study considers descriptive features along with a large set of features constructed from heart rate and accelerometer measurements collected during submaximal running on a treadmill. Then, it employs a data-driven approach to select a small number of the most predictive features to include in a linear regression model for predicting VO_2max_. Furthermore, we evaluate how the performance is affected by combining heart rate features and accelerometer features compared to using these features separately.

## Data collection

### Subjects

A sample of convenience including 31 recreational runners (15 men and 16 women aged 19-26 years) volunteered to participate in this study. Subjects were recruited during March 2016 via local advertisements and flyers, and were invited to participate via e-mail correspondence if they met the inclusion-exclusion criteria of the study. Only subjects who had been running regularly and had prior experience with treadmill running were eligible to be included in the study. All subjects had no self-reported history of metabolic, neurological, pulmonary, or cardiovascular disease or surgery to the back or lower limbs. Furthermore, all were symptom-free of any lower extremity injury for at least six months prior to the study. All runners provided written informed consent prior to participation in accordance with the Declaration of Helsinki. The local ethics committee of Stellenbosch University approved the study (#SU-HSD-002032).

20 of the initial 31 who participated in the first VO_2max_ test volunteered to participate in a second VO_2max_ test after undergoing a supervised eight-week training intervention designed to improve the aerobic capacity of running. During this intervention, there were seven dropouts (five due to running-related injury and two due to lack of training adherence). Thus a total of 13 subjects (four men and nine women) were able to perform a second VO_2max_ test post intervention. In our analysis, we included the first test of all participants, as well as the second one if it was performed. As a runner’s VO_2max_ can change as a result of training activities, we tried to incorporate this type of variability in our dataset by including both tests.

### Protocol

**Fig 1:**
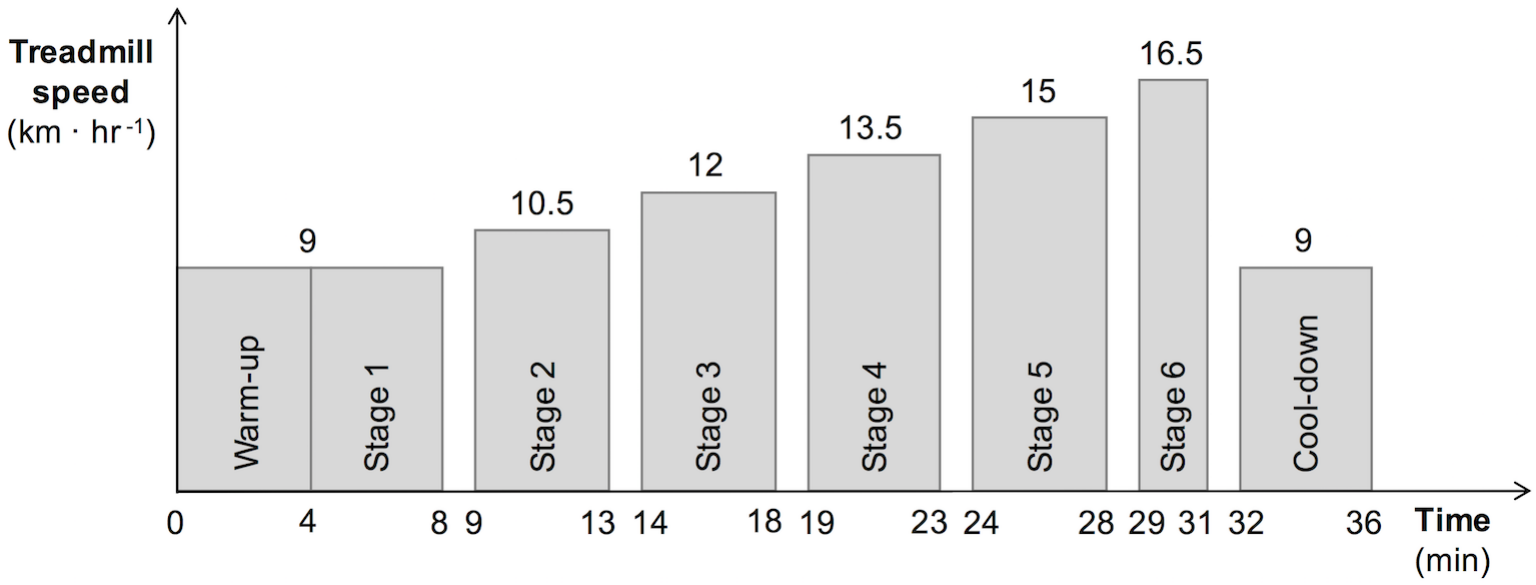
Example of the protocol for a male runner reaching stage 6.

Each subject performed one or two maximal incremental running tests to exhaustion on a motorized treadmill (Saturn h/p/cosmos, Nussdorf-Traunstein, Germany). If two tests were performed, the second one always took place at least seven weeks after the first test. An example of the protocol is shown in Fig 1. The test began with a four minute warm-up, at a running speed of 8 km ⋅ hr^−1^ for women and 9 km ⋅ hr^−1^ for men. After the warm-up, the test proceeded with four minute stages, each of which was followed by one minute of rest, until volitional exhaustion. The first stage employed the same running speed used during the warm-up, and each new stage saw the treadmill speed increase discontinuously in increments of 1.5 km ⋅ hr^−1^. The treadmill gradient was fixed at 1% throughout the submaximal assessments to reflect the energetic cost of outdoor running [25]. Participants could run in their own relatively new (within three months of use) conventional shod running shoes. All tests were performed under similar laboratory conditions (20-25°C, 50-60% relative humidity, and an altitude of 130m). Participants were fitted with an adjustable safety harness during the entire treadmill test. Each subject reported a rating of perceived exertion score [26] immediately after each stage. Runners were considered to have achieved VO_2max_ when at least two of the following criteria were fulfilled:

1. a plateau in the oxygen uptake (VO_2_) as defined by an increase of less than 1.5 ml ⋅ kg^−1^ ⋅ min^−1^ in two consecutive stages;
2. a respiratory exchange ratio (RER) > 1.15;
3. a maximal heart rate value (HR_max_) > 95% of the age-predicted maximum (220 − age);
4. a rating of perceived exertion (RPE) ≥ 19 on the 6-20 Borg scale.

All tests were terminated by volitional exhaustion, and all subjects achieved VO_2max_ by the set criteria. Specifically, all subjects met the first and second criteria (VO_2_ plateau; RER > 1.15), while three subjects failed to meet the third criterion (one with a faulty HR reading and two with a HR_max_ of 90% and 92% respectively), and two failed to meet the fourth criterion (RPE of 18 and 18.5 respectively).

For the analysis, six treadmill tests were excluded from the dataset. The heart rate measurements of four tests showed an irregular pattern that was probably caused by a poorly connected heart rate strap. In two other tests, the accelerometer data failed to record. Table 1 shows the descriptive characteristics of the participants for the remaining treadmill tests.

**Table 1:** Descriptive characteristics of the subjects. Notation: mean ± SD.

	Men	Women	All
Number of subjects	12	16	28
Number of tests	16	25	41
Age (years)	22.06 ± 2.11	21.56 ± 0.70	21.76 ± 1.44
Height (m)	1.79 ± 0.07	1.68 ± 0.06	1.72 ± 0.08
Body mass (kg)	75.34 ± 11.23	61.55 ± 7.18	66.93 ± 11.22
BMI (kg ⋅ m^−2^)	23.46 ± 2.85	21.84 ± 2.38	22.47 ± 2.69
VO_2max_ (ml ⋅ kg^−1^ ⋅ min^−1^)	51.55 ± 5.74	43.65 ± 4.90	46.73 ± 6.51

### Measurements

In this section, we describe the data measured during the treadmill tests that will be used to calculate VO_2max_ and the features for the prediction models.

#### Oxygen uptake

The pulmonary gas exchange was recorded throughout the incremental test using a breath-by-breath metabolic analyzer (Cosmed Quark CPET, Rome, Italy). The gas analyzers were calibrated before each session to 16% O_2_, 4% CO_2_ balance N_2_ and the turbine flow meter was calibrated with a 3L calibration syringe before each test. Oxygen uptake (VO_2_) was calculated from the O_2_ measurements divided by body mass. For each treadmill test, the maximal oxygen uptake (VO_2max_) of the runner was calculated as the maximum value of the rolling average of the VO_2_ signal with a window length of 30 seconds.

#### Heart rate

During each treadmill test, the subject’s heart rate (HR) was sampled breath-by-breath according to the gas exchange using a heart rate monitor (Cosmed Quark CPET, Rome, Italy). The samples were then averaged every 10 seconds. As the averaged signal was often still noisy, small fluctuations and sudden peaks were removed by smoothing the signal using a median filter, where each measurement *x*_*t*_ was replaced by the median of {*x*_*t*−3_, …, *x*_*t*_, …, *x*_*t*+3_}.

#### Acceleration

**Fig 2:**
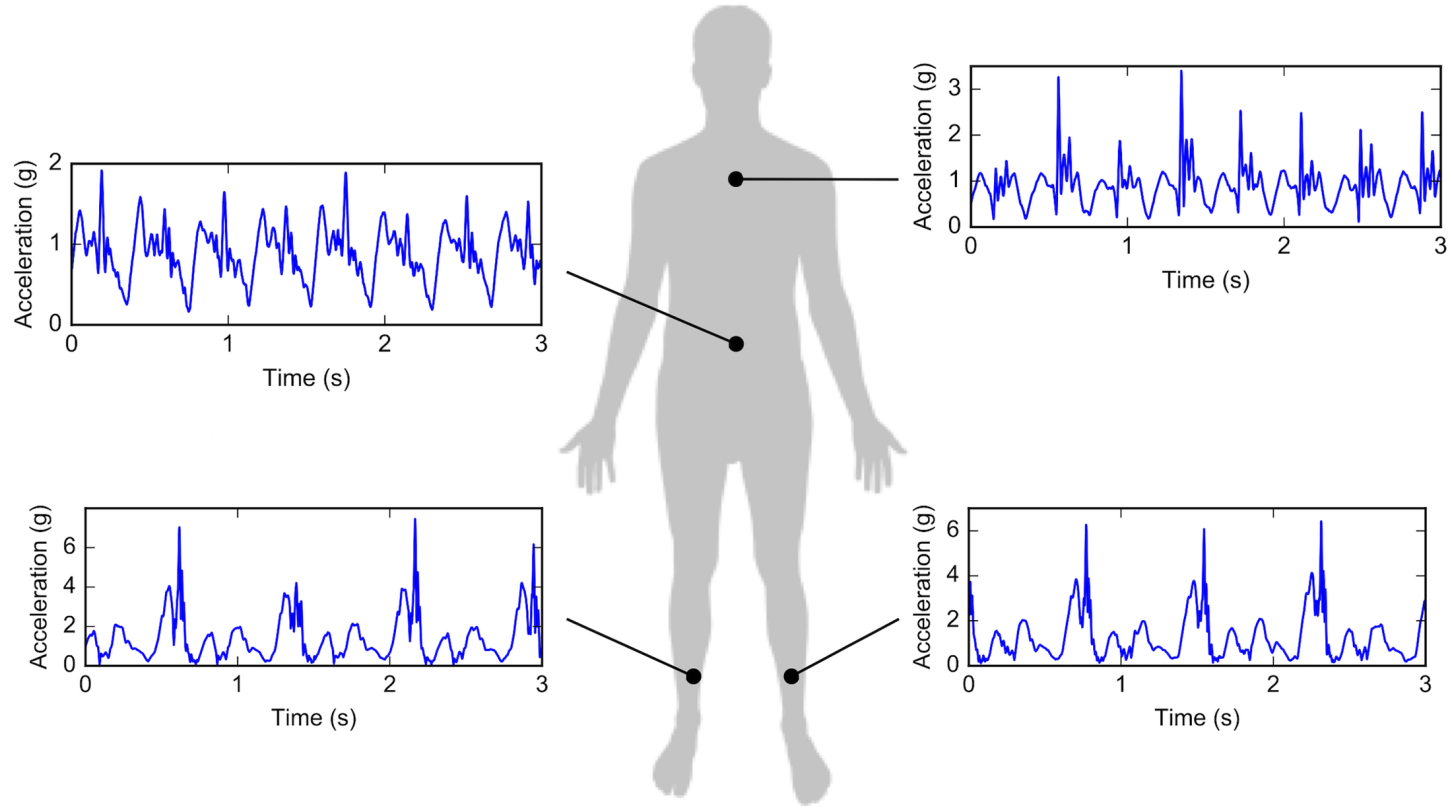
Locations of the accelerometers attached to the runners’ bodies. For each location, an example signal of the total acceleration over three seconds is shown. Note that only one of the two tibia accelerometers is used in this study.

Acceleration was measured using wearable inertial measurement units (Shimmer3 wireless IMU, sampling rate 1024Hz, range ±16g, Dublin, Ireland) at four locations: upper back, lower back, and left and right tibia, as shown in Fig 2. The upper back accelerometer was aligned between the shoulder blades at the level of the C7-T2 spinal processes. The lower back accelerometer was aligned between the posterior superior iliac spines at the level of the L3-L5 spinal processes, and the tibial accelerometers were aligned on the antero-medial aspect of the distal tibia, 8cm above the medial malleolus. In two tests, one of the tibia accelerometers fell off. Therefore only data from one tibia accelerometer is used. The right one is used in the one trial where the left one fell off, and the left one is used for the remaining 40 trials.

The accelerometer measurements were sampled at 1024 Hz. To remove noise, the acceleration signals were filtered using a low-pass filter with a cut-off frequency of 50 Hz, which is high enough to capture characteristics of running patterns. To make sure that the axes of the accelerometers were rotated correctly, the Moe-Nilssen tilt correction method [27] was used to align the axes with the anterior-posterior, mediolateral, and vertical direction of the runners. This method also subtracts the static gravity component (1g) from the vertical acceleration.

## Experiments

We perform two experiments on the data collected during the treadmill test. In the first experiment, we explore a data-driven approach to find a good feature set by comparing different combinations of descriptive, heart rate and accelerometer features. The best combination found in this experiment will serve as our final model. In the second experiment, we replicate Weyand et al.’s model [14] using the sensors available in our study and compare its performance to our best model.

### Experiment 1: Our approach

#### Feature extraction

Three types of features are used in this study: descriptive features, heart rate features and accelerometer features. Since the goal is to develop a model for predicting VO_2max_ from submaximal exercise, the latter two types of features are extracted from the warm-up stage and the first three stages of the test only. The third stage was performed at 12 km ⋅ hr^−1^ for men and 11 km ⋅ hr^−1^ for women, and it was confirmed that it represented submaximal running by the respiratory exchange ratio being < 1. Table 2 summarizes the features, which are described in more detail next.

**Table 2:** Overview of all features.

Category	#	Features	
**Descriptive**	2	Gender (0 = male, 1 = female)	G
		Body mass (kg)	BM
**Heart rate**	8	Average	${\text{HR}}_{i}^{\left(-1\right)}$
**Acceleration**	480	Average	${\text{HR}}_{l,d,i}^{\left(-1\right)}$
		Standard deviation	${\text{SD}}_{l,d,i}^{\left(-1\right)}$
		Variance	${\text{VAR}}_{l,d,i}^{\left(-1\right)}$
		Root mean square	${\text{RMS}}_{l,d,i}^{\left(-1\right)}$
		Power	$P_{l,d,i}^{\left(-1\right)}$

Notation: stage number *i* = 0, 1, 2, 3 (stage 0 is the warm-up stage); accelerometer location *l* = t, bl, bu (t = left or right tibia, bl = lower back, bu = upper back); direction *d* = *x*, *y*, *z*, total (*x* = anterior-posterior, *y* = mediolateral, *z* = vertical); the superscript (−1) means that both the feature and its inverse are computed.

All models listed in the overview of Abut et al. [4] use (a subset of) gender, body mass, length, BMI and age as descriptive features. This study considers two of these features: gender (G: 0 = male, 1 = female) and body mass (BM) in kg, which are known to be relevant for predicting VO_2max_. Given the relatively small age range of the subjects (19-26 years) and that VO_2max_ decreases approximately 0.2-0.5 ml ⋅ kg^−1^ ⋅ min^−1^ per year [28], age is not considered.

From the heart rate measurements we calculate the average heart rate (HR) for each stage of the test. Like Weyand et al. [14], we also calculate the inverse of the average heart rate because of the inverse relation between heart rate and maximal oxygen uptake [29]. Because the heart rate dropped during the rest periods, the average is computed only over the last minute of each stage where the heart rate was more stable.

From the accelerometer data, the following five features are extracted from each stage: average (AVG), standard deviation (SD), variance (VAR), root mean square (RMS) and power (P). RMS is often used in studies related to running gait analysis [30, 31]. The other features are commonly used to describe movement patterns based on accelerometer measurements. Similarly to the heart rate features, we also compute the inverse of each feature, since the accelerometer features may have an inverse relation to VO_2max_ as well. Each feature is calculated for the anterior-posterior (*x*), mediolateral (*y*), vertical (*z*) and total ($\sqrt{x^{2}+y^{2}+z^{2}}$) acceleration signals measured at the upper back, lower back and left tibia (or right tibia if the accelerometer on the left tibia fell off). Because the treadmill accelerated and decelerated at the start and end of each stage, the first and last ten seconds of each stage are discarded.

#### Prediction method

We employ a mixed-effects unpenalized linear regression model to predict VO_2max_. We chose a linear model because it offers reasonable interpretability. This is important for sports scientists, coaches and athletes who want to gain insight into which features influence the prediction. Moreover, linear models tend to offer more robustness against overfitting for small sample sizes, provided that a relatively small feature set is used, which we ensure by performing feature selection as described in the following section.

Some subjects performed two trials (before and after an intervention) while others completed only one trial. To account for the potential correlation between repeated observations, we use a mixed-effects model where the variable ‘Test’ (which has the value ‘pre’ or ‘post’ depending on whether the trial was performed before or after the intervention, respectively) is a random effect for the intercept. We use the *lme4* package [32] in R and specify the regression formula as follows:

$$\begin{array}{c} \begin{array}{c} {\text{VO}}_{2\text{max}}∼1+\left(1|\text{Test}\right)+G+\text{BM}+... \end{array} \end{array}$$

where G, BM, … are the fixed-effect variables included in the model, which are selected using the feature selection method described in the next section.

#### Feature selection

We combine the descriptive, heart rate and acceleration features (see Table 2) into one feature set. However, given that the sample size is 41 data points, including all 490 features in the model may result in overfitting. Therefore, we select a subset of the features using a variant of greedy forward selection [33]. Greedy forward selection is a wrapper-based approach that typically starts with an empty feature set. It then iteratively adds the single best feature from a candidate set to the feature set until some stopping criterion is satisfied.

Here, instead of starting from an empty feature set, we begin with a feature set *F* that contains the two descriptive features. All the heart rate and accelerometer features are added to the set of candidate features *C*. In each iteration, we assess the quality of each feature *f* ∈ *C* by learning a linear regression model *M*′ using the feature set *F*′ = *F* ∪ {*f*} as input. We evaluate *f*’s quality by using internal leave-one-subject-out cross-validation on the training data to calculate *M*′’s adjusted explained variance ($R_{\text{adj}}^{2}$):

$$\begin{array}{c} \begin{array}{cc} R_{\text{adj}}^{2} & =1-\frac{n-1}{n-p-1}\cdot \left(1-R^{2}\right)=1-\frac{n-1}{n-p-1}\cdot (\frac{\sum_{i=1}^{N}{\left({\hat{y}}_{i}-y_{i}\right)}^{2}}{\sum_{i=1}^{N}{\left(y_{i}-\bar{y}\right)}^{2}}) \end{array} \end{array}$$

where *n* is the number of instances used to train the model and *p* is the number of features. We use $R_{\text{adj}}^{2}$ instead of R^2^ because it corrects for the fact that *F*′ has a different number of features than *F*. In each iteration of the forward selection, the highest scoring feature *f*_*b*_ is added to *F* (i.e., *F* = *F* ∪ {*f*_*b*_}) and removed from *C* (i.e., *C* = *C* \ {*f*_*b*_}) provided that adding *f*_*b*_ to *F* results in an improvement of at least 0.05 in the $R_{\text{adj}}^{2}$. We use this improvement threshold as an additional countermeasure against overfitting. The selection process is terminated when no feature meets the improvement threshold.

#### Experimental set-up

We compare four different combinations of descriptive features, heart rate features and accelerometer features:

F_1_: uses only the two descriptive features: gender and body mass;
F_2_: combines F_1_ with the heart rate features;
F_3_: combines F_1_ with the accelerometer features;
F_4_: combines F_1_ with both the heart rate and the accelerometer features.

### Experiment 2: Replicating Weyand et al.’s model

Weyand et al. [14] proposed a model to predict VO_2max_ based on the ratio $t_{c}^{-1}/\text{HR}$, where *t*_*c*_ is the foot-ground contact time and HR is the average heart rate as measured over several minutes of running. This study found that these variables show a linear and parallel increase as the running speed increases, and that the ratio $t_{c}^{-1}/\text{HR}$ is related to VO_2max_. In Weyand et al.’s study, contact time was measured via an accelerometer placed on the foot. Next, we describe how we compare to Weyand et al.’s model given that we do not have access to foot-based accelerometer data.

#### Feature extraction

**Fig 3:**
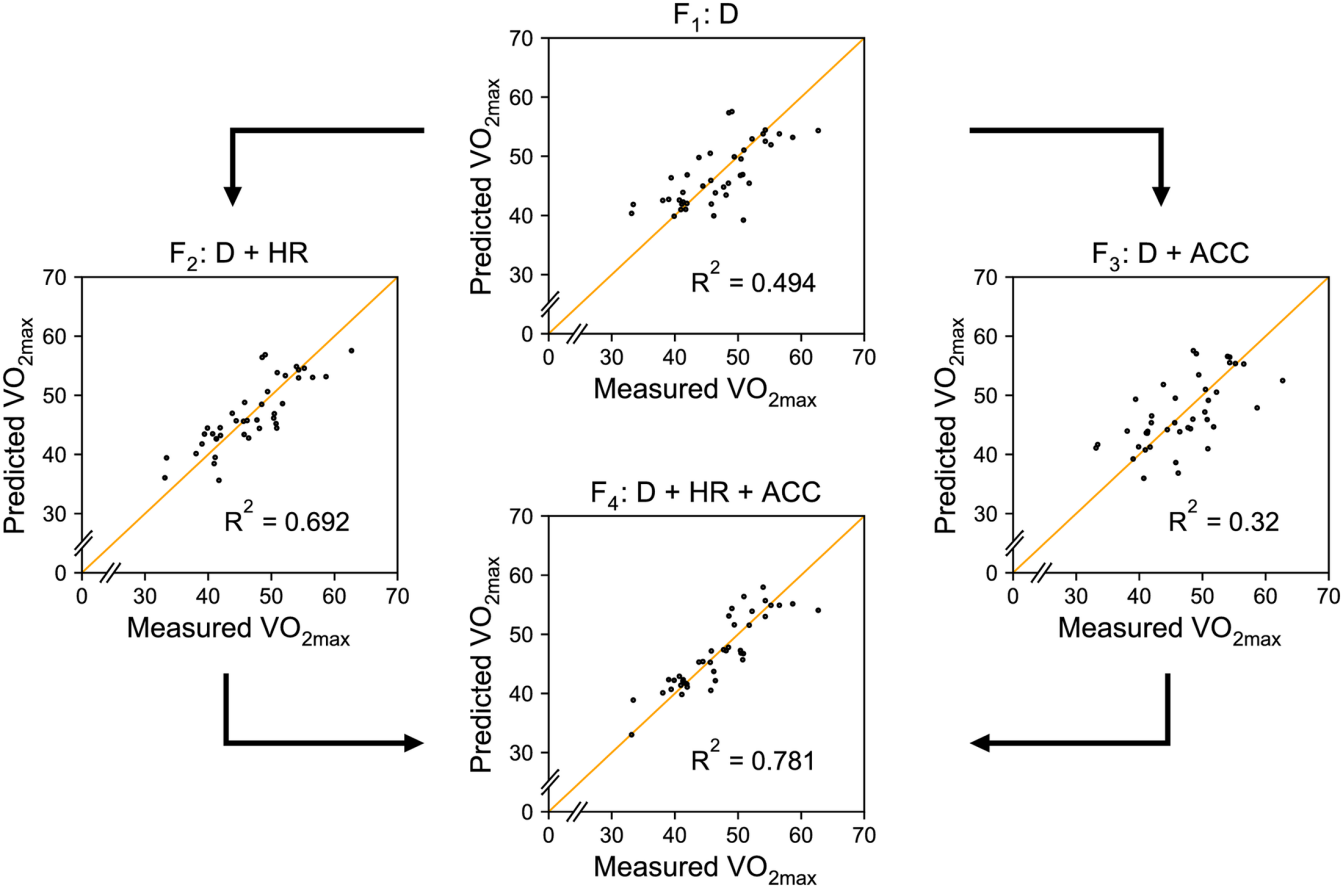
Calculation of contact time from the vertical lower back acceleration. The green and red dots indicate respectively starts and ends of foot-ground contact.

We employ Gaudino et al.’s method [34] for calculating the contact time from the vertical acceleration at the center of mass (COM). Since the lower back accelerometer is positioned close to the COM during running, we use this accelerometer to estimate contact time. The start and end of foot-ground contact is determined by detecting where the signal crosses zero, as shown in Fig 3. To identify these points, the signal is first smoothed using a 4^th^-order Butterworth low-pass filter with a cut-off frequency of 15Hz. For each of the first three stages of the treadmill test, we estimate the contact time ($t_{c}^{i}$) as the average over all steps within stage *i*. We also compute the average heart rate (*HR*^*i*^) from the last minute of stage *i* as previously described. We then calculate ${\left(t_{c}^{i}\right)}^{-1}/{\text{HR}}^{i}$ for each stage and average the three values to obtain the value of the final ratio feature.

Some differences exist in the way we compute the ratio feature compared to Weyand et al.’s study. First, we compute the average contact time for each stage ($t_{c}^{i}$) using the complete stage. In Weyand et al.’s study, $t_{c}^{i}$ is computed using ≥ 20 consecutive steps at least 30 seconds into the stage. Second, we calculate the average heart rate (*HR*^*i*^) using the last minute of the stage whereas Weyand et al. compute *HR*^*i*^ as the average of the heart rate values measured at 3.75, 4.75 and 5.25 minutes after the start of the stage. Because Weyand et al.’s protocol does not have a warm-up stage while our protocol does, we omit the data from the warm-up stage in this comparison.

#### Experimental set-up

Weyand et al. proposed two approaches based on the ratio $t_{c}^{-1}/\text{HR}$ combined with the gender of the subjects. The first learned separate linear regression models for men and women, each of which used only $t_{c}^{-1}/\text{HR}$ as input. The second learned a single linear regression model using both gender and $t_{c}^{-1}/\text{HR}$ as inputs. We evaluate both approaches and compare the results to our method. Note that we employ a fixed-effects linear model here to keep the set-up similar to Weyand et al.’s study.

## Evaluation

Given the small sample size, we use leave-one-subject-out cross-validation to evaluate the models. In this cross-validation scheme, the data of one subject (one or two treadmill tests) are used as test data while the data of the other subjects are used for selecting features and training the model. This means that the features of the model are selected separately for each subset. Hence, in the feature selection process, the $R_{\text{adj}}^{2}$ values to evaluate features are computed using an *inner* cross-validation loop, while the models are evaluated using an *outer* cross-validation loop.

The predicted VO_2max_ values are evaluated using the following metrics: the explained variance (R^2^) of the model, the mean absolute error (MAE) and the root mean squared error (RMSE) expressed in ml ⋅ kg^−1^ ⋅ min^−1^. We also report the mean absolute percentage error (MAPE) and the root mean squared relative error (RMSRE). These evaluation metrics are defined as follows:

$$\begin{array}{c} \begin{array}{cc} R^{2} & =1-\frac{\sum_{i=1}^{N}{\left({\hat{y}}_{i}-y_{i}\right)}^{2}}{\sum_{i=1}^{N}{\left(y_{i}-\bar{y}\right)}^{2}}\\ \text{MAE} & =\frac{1}{N}\cdot \sum_{i=1}^{N}\left|{\hat{y}}_{i}-y_{i}\right|\\ \text{MAPE} & =\frac{1}{N}\cdot \sum_{i=1}^{N}|\frac{{\hat{y}}_{i}-y_{i}}{y_{i}}|\\ \text{RMSE} & =\sqrt{\frac{1}{N}\cdot \sum_{i=1}^{N}{\left({\hat{y}}_{i}-y_{i}\right)}^{2}}\\ \text{RMSRE} & =\sqrt{\frac{1}{N}\cdot \sum_{i=1}^{N}{\left(\frac{{\hat{y}}_{i}-y_{i}}{y_{i}}\right)}^{2}} \end{array} \end{array}$$

where *y* are the measured VO_2max_ values (with average $\bar{y}$) and $\hat{y}$ are the predicted values for the *N* = 41 treadmill tests.

## Results

### Results for experiment 1: Data-driven model selection

**Fig 4:**
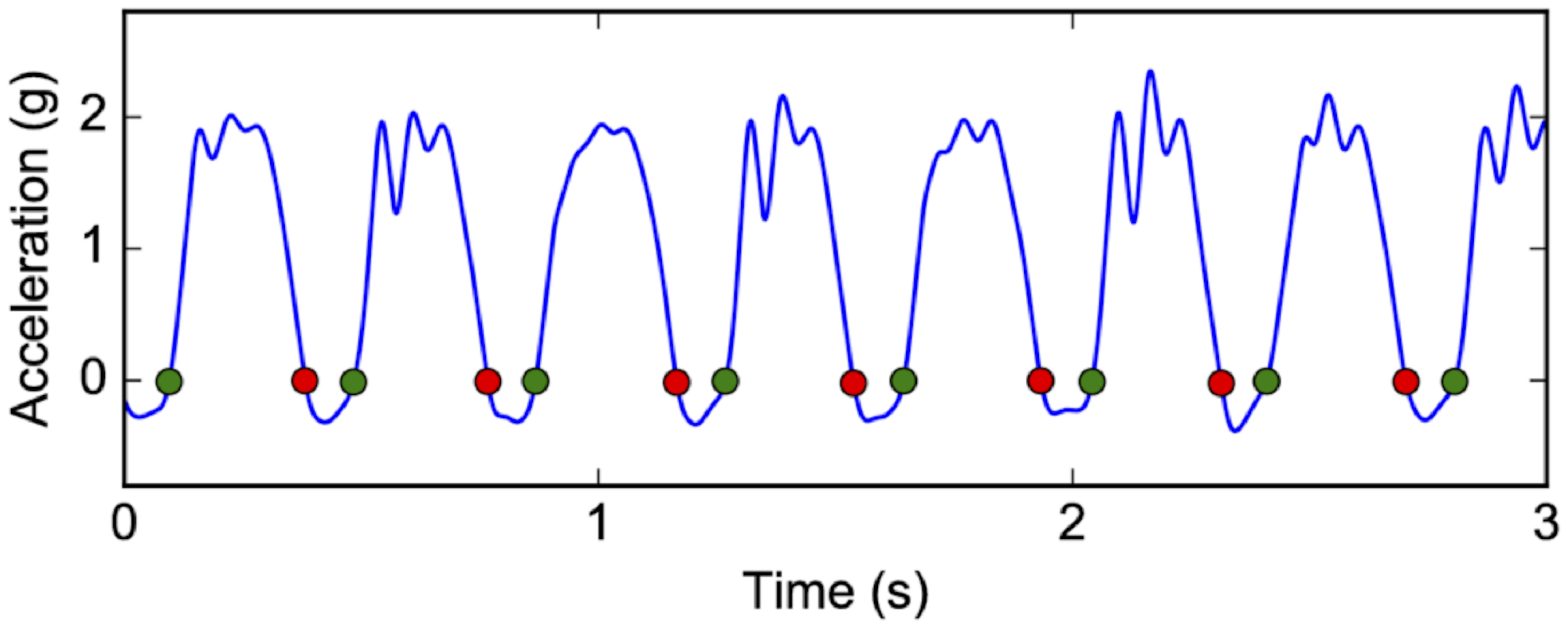
Measured vs predicted VO_2max_ values. D = descriptive features, HR = heart rate features, ACC = accelerometer features. Points that are closer to the orange line, on which the measured VO_2max_ equals the predicted VO_2max_, correspond to more accurate predictions.

Fig 4 shows how the predicted VO_2max_ values fit the measured values for each of the four feature set combinations: F_1_, F_2_, F_3_ and F_4_. The VO_2max_ values are predicted using leave-one-subject-out cross-validation, where in each fold we first select features using the training data, and then learn a mixed-effects linear regression model using the same training data again. The supporting tables (S1–S3 Tables) show the number of folds that each feature was selected in when using feature sets F_2_, F_3_ and F_4_, respectively. Note that no feature selection is used for F_1_: gender and body mass are always included.

Table 3 summarizes the results for each combination according to all five evaluation metrics. F_4_, the combination of descriptive features, heart rate and accelerometer features, results in a mean absolute error of 2.33 ml ⋅ kg^−1^ ⋅ min^−1^. In percentage terms, the average prediction error is 4.92%, meaning that the predicted VO_2max_ is on average within 5% of the true VO_2max_ value. Additionally, this model has an explained variance (R^2^) of 0.781, which is better than all other combinations. Regardless of the metric, the models are ranked in the same order: F_4_ > F_2_ > F_1_ > F_3_. This shows that the accelerometer data improve the predictions, but only if used in combination with the heart rate data.

**Table 3:** Comparison of the four combinations.

Combination	R^2^	MAE	MAPE	RMSE	RMSRE
F_1_	0.494	3.53	7.75%	4.63	10.33%
F_2_	0.692	2.98	6.51%	3.61	7.91%
F_3_	0.320	4.24	9.28%	5.363	11.87%
F_4_	0.781	2.33	4.92%	3.05	6.34%

R^2^ = explained variance, MA(P)E = mean absolute (percentage) error and RMS(R)E = root mean squared (relative) error. MAE and RMSE are expressed in ml ⋅ kg^−1^ ⋅ min^−1^.

Table 4 shows the fixed-effect coefficients for each of the four combinations, inferred using the full dataset. In the best combination (F_4_) four features were selected: gender, body mass, the inverse of the average heart rate during the warm-up stage (${\text{HR}}_{0}^{-1}$) and the inverse of the variance of the total tibia acceleration during the warm-up stage (${\text{VAR}}_{t,\text{total},0}^{-1}$).

**Table 4:** Predictor functions. Fixed-effect coefficients learned from the complete dataset.

Combination	Function
F_1_	79.64 − 13.04 × G − 0.3728 × BM
F_2_	$43.77-9.741\times G-0.3182\times \text{BM}+4381\times {\text{HR}}_{0}^{-1}$
F_3_	77.58 − 9.769 × G − 0.2676 × BM − 14.41 × RMS_bl,y,1_
F_4_	$25.78-8.861\times G-0.2538\times \text{BM}+5546\times {\text{HR}}_{0}^{-1}+4.879\times {\text{VAR}}_{t,\text{total},0}^{-1}$

G = gender, BM = body mass, ${\text{HR}}_{0}^{-1}$ = inverse of the average heart rate of the warm-up stage, RMS_bl,y,1_ = root mean squared acceleration of the mediolateral lower back acceleration in stage 1, ${\text{VAR}}_{t,\text{total},0}^{-1}$ = inverse of the variance of the total tibia acceleration in the warm-up stage.

### Results for experiment 2: Comparison to Weyand et al.

**Fig 5:**
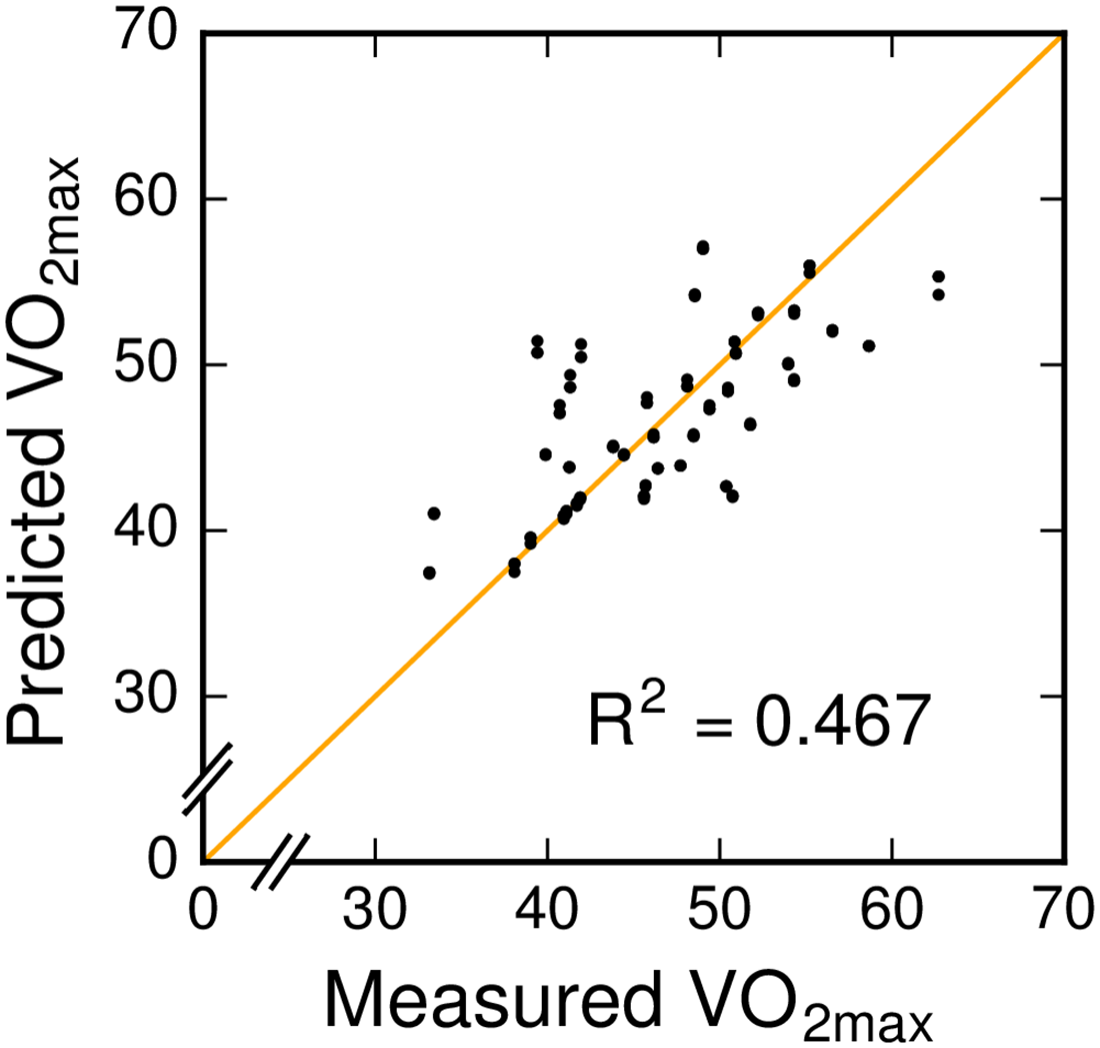
Evaluation of the second method of Weyand et al. using gender and $t_{c}^{-1}/\text{HR}$ as features.

Table 5 shows the results for all five metrics for both models. The first method results in a MAE of 3.65 ml ⋅ kg^−1^ ⋅ min^−1^ (or relative terms 7.96%) and an explained variance (R^2^) of 0.441. Like in Weyand et al.’s study, the second method performs better with a MAE of 3.58 ml ⋅ kg^−1^ ⋅ min^−1^ (or relative terms 7.81%) and an R^2^ value of 0.467. The model fit for the second method is shown in Fig 5.

**Table 5:** Comparison of the two methods of Weyand et al.

Method	R^2^	MAE	MAPE	RMSE	RMSRE
1	0.441	3.65	7.96%	4.86	10.89%
2	0.467	3.58	7.81%	4.74	10.63%

R^2^ = explained variance, MA(P)E = mean absolute (percentage) error and RMS(R)E = root mean squared (relative) error. MAE and RMSE are expressed in ml ⋅ kg^−1^ ⋅ min^−1^.

## Discussion

### Combining heart rate and accelerometer features

The results indicate that the data-driven approach employed in this study can be used to automatically find relevant features to predict VO_2max_. The comparison of the different models in Fig 4 shows that features derived from body-worn technology improve the predictions compared to only considering descriptive features. The integration of physiological and biomechanical systems further improves the model. While the related work by Weyand et al. [14] and Tönis et al. [15] is based on the same idea, we show that considering a broader set of features derived from accelerometer measurements may be beneficial for predicting VO_2max_.

**Fig 6:**
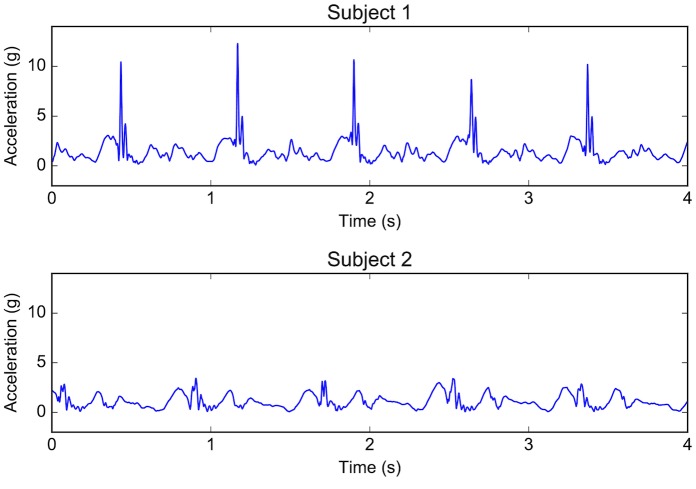
Total tibia acceleration of two similar subjects. The two subjects have the same gender (G = 1 = female), a similar body mass (BM = 71.8 kg for subject 1 and 68.5 kg for subject 2), and a similar inverse heart rate in the warm-up stage (${\text{HR}}_{0}^{-1}$ = 0.00559 for subject 1 and 0.00557 for subject 2). While the value of the ${\text{VAR}}_{t,\text{total},0}^{-1}$ feature is low for subject 1 (0.689), it is high for subject 2 (2.30). Consequently, subject 1 has a lower VO_2max_ (33.14 ml ⋅ kg^−1^ ⋅ min^−1^) than subject 2 (41.71 ml ⋅ kg^−1^ ⋅ min^−1^).

The best prediction model found in this study is based on four features: gender, body mass, the inverse of the average heart rate of the warm-up stage (${\text{HR}}_{0}^{-1}$) and the inverse of the variance of the total tibia acceleration in the warm-up stage (${\text{VAR}}_{t,\text{total},0}^{-1}$). The first two features are known to be related to VO_2max_ and are used in most existing models. The third feature is the inverse of the average heart rate and represents the inverse relation between heart rate and VO_2max_ [29]. The last feature is the inverse of the variance of the total tibia acceleration (${\text{VAR}}_{t,\text{total},0}^{-1}$). To gain insight into how to interpret this feature, we compare the total tibia acceleration in the warm-up stage of two subjects with the same gender, a similar body mass and a similar value for the ${\text{HR}}_{0}^{-1}$ feature. Fig 6 shows four seconds of each subject’s signals. The subject with the higher VO_2max_ (subject 2) has a higher value of ${\text{VAR}}_{t,\text{total},0}^{-1}$ which corresponds to a lower variance of the total tibia acceleration signal. Since this signal includes the entire gait cycle, the 3D accelerations generated during both the swing phase (i.e., movement) and the contact phase (i.e., ground reaction forces) contribute to the value of the feature. As can be seen from this comparison, the difference in the variance is mainly caused by the height of the peaks generated during the contact phase. The relation of this feature to running VO_2max_ is interesting as it has not been used before for the prediction of VO_2max_ from submaximal running.

### Replicating Weyand et al.’s model

Compared to Weyand et al.’s paper, we report a more comprehensive set of error metrics. According to all five metrics, our learned model using F_4_ results in more accurate predictions than using Weyand et al.’s model based on our available sensors. While our best model obtained a better R^2^ than either of Weyand et al.’s models as reported in their paper, our replication did result in lower R^2^ values than was reported in the original paper. There are several possible explanations for this. First, we use an accelerometer placed on the lower back instead of on the foot to estimate contact time. Calculating the contact time using a lower back accelerometer may be less accurate and hence these errors may negatively influence the predictions of the model. Second, all subjects of the present study are recreational runners of 19-26 years old, while some participants in Weyand et al.’s study [14] ran > 1 hour each day and the oldest runner was 47 years old. These differences may affect the generalizability of the model to new data. Third, our study used a different protocol. In Weyand et al.’s protocol [14], subjects ran in bouts of 5.5 min, with rest intervals of 3-5 min. In contrast, the subjects in our protocol ran in stages of 4 min with rest intervals of 1 min. These protocol differences affect heart rate due to recovery and thus the predictions as well.

### Practical use of the model

**Fig 7:**
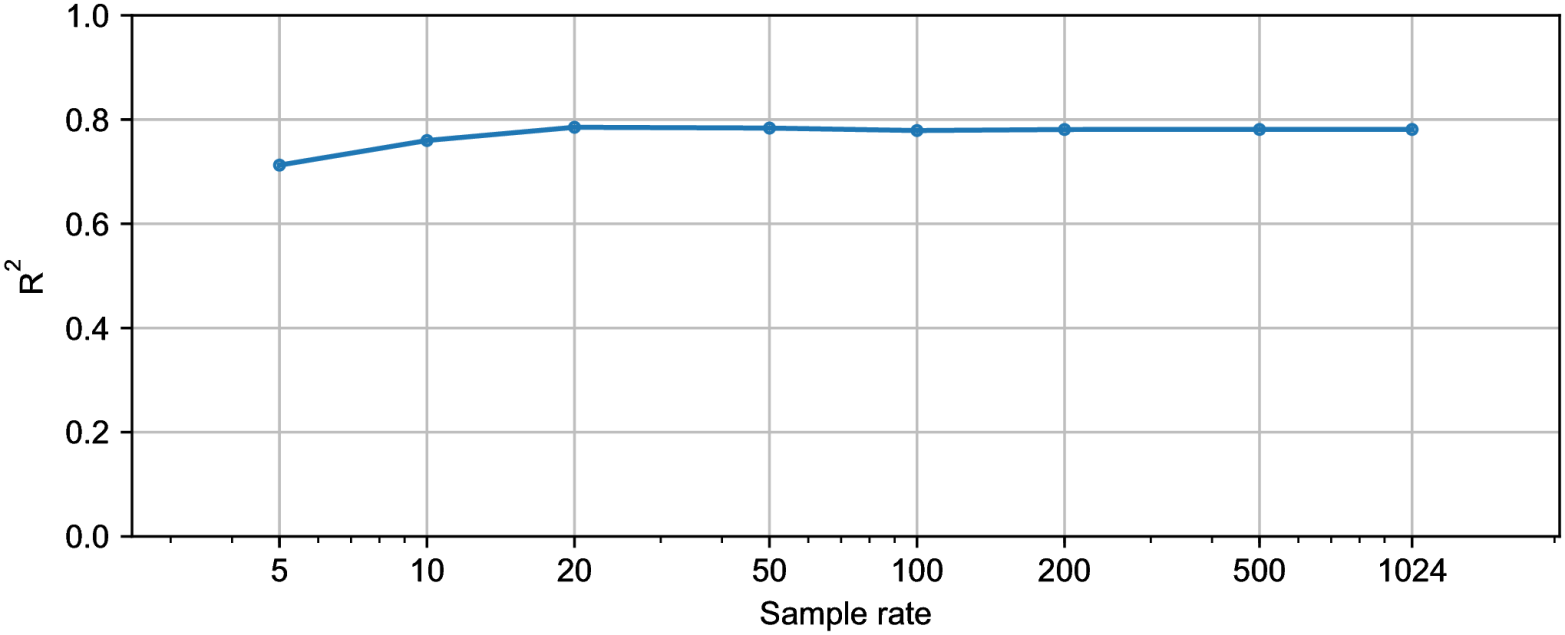
Explained variance (R^2^) of F_4_ when down-sampling the tibia acceleration to different sample rates.

Two sensors are required in the final model: a heart rate monitor and an accelerometer attached to the left or right tibia. While most runners currently use a sports watch equipped with a heart rate monitor, the use of the tibia accelerometer may be less practical. More specifically, three aspects should be considered. First, the tibia accelerometer should be firmly attached so that it does not fall off as occurred in one test in this study. A practical tool therefore needs a compact and lightweight device. Second, the accelerometer should be attached at the correct position, which is the antero-medial aspect of the distal tibia. One possibility is to embed the device in the clothing of the athlete. Third, commercially available accelerometers typically have a lower sampling rate than 1024 Hz, which was used in this study. The sample rate may affect the values of the features computed from the accelerometer signal, and hence the predictions of the model. To check the robustness of the predictions to this factor, we calculated the value of the tibia feature for each test example from a down-sampled acceleration signal. We then evaluated the model, which was trained using the 1024 Hz data, for the down-sampled data. Fig 7 shows the R^2^ when using F_4_ as a function of the sample rate. These results show that using commercially available accelerometers, which can usually sample accelerations at ≥ 50 Hz, will not decrease the model’s explained variance.

Another practical aspect is the speed at which subjects need to run in order to compute the features extracted from the sensors. Since this study’s goal was to develop a submaximal exercise model, lower running speeds are preferred. Both the selected heart rate feature and the accelerometer feature are computed during the warm-up phase, which means that an individual’s VO_2max_ could be predicted from only four minutes of running at a speed of 8 or 9 km ⋅ hr^−1^. As this is a low exercise intensity, athletes could regularly estimate their VO_2max_ to closely monitor training adaptations.

A final practical consideration is that the model in this paper is based on running at fixed velocities on a treadmill. As most acceleration-based features are speed dependent, our model may not be applicable to data collected during outdoor running, where the running speed varies. However, the same data-driven approach presented in this paper could be applied to data from outdoor running to develop a model capable of predicting VO_2max_ in that setting.

### Generalizability

The use of leave-one-subject-out cross-validation means that the error estimates evaluate the model’s ability to generalize to unseen individuals who have similar characteristics to the subjects in our data sample. However, an unseen individual may differ in two important ways from the subjects in this study. First, all participants of this study were recreational runners. It is unclear how well this model would translate to elite athletes who have higher VO_2max_ values. Additional research would be needed to ascertain if VO_2max_ can be predicted accurately from submaximal effort for elite athletes. Second, all participants were between 19 and 26 years old. Given that VO_2max_ decreases approximately 0.2-0.5 ml ⋅ kg^−1^ ⋅ min^−1^ per year [28], the quality of the model’s predictions will likely be lower for younger or older individuals. If the age range of a study’s participants is wider, then including age as a feature in the model may be valuable.

## Conclusion and future work

In this study, we have shown that VO_2max_ can be predicted from a combination of descriptive features, heart rate features and accelerometer features derived from data collected during submaximal running. We defined a large set of features based on the sensor data and employed a data-driven approach to select a small subset of them to include in a mixed-effects linear regression model. We evaluated the benefit of each category of features (descriptive, heart rate, and accelerometer) and found that considering all three types resulted in the best performance. The best model found in this paper had an explained variance of 0.781 and used four features: two descriptive features (gender and body mass), one heart rate feature (${\text{HR}}_{0}^{-1}$) and one accelerometer feature (${\text{VAR}}_{t,\text{total},0}^{-1}$). This model can predict an individual’s VO_2max_ from objective variables calculated from running on a treadmill at only 8 or 9 km ⋅ hr^−1^ for four minutes.

There are two limitations to the model. First, as the participants in our study were recreational runners between 19 and 26 years of age, the model is likely not applicable for elite runners and subjects outside of this age range. An interesting future direction would be developing models to predict the VO_2max_ from elite athletes, as well as considering subjects with a wider range of ages. Second, our model is based on running activity on a treadmill. In future work, it would be interesting to investigate predicting VO_2max_ based on outdoor running.

## Supporting information

S1 Table Selected features for F_2_. (PDF)

S2 Table Selected features for F_3_. (PDF)

S3 Table Selected features for F_4_. (PDF)
