# Supplementary material for: Data fusion of body-worn accelerometers and heart rate to predict VO2max during submaximal running
Source: PLoS One. 2018 Jun 29;13(6):e0199509. doi: 10.1371/journal.pone.0199509 (PMC6025864; doi:10.1371/journal.pone.0199509)
Supplement: S2 Table — (PDF) [file pone.0199509.s002.pdf]

S2 Table. Selected features for F_3. There were 28 folds in the leave-one-subject-out cross-validation. This table shows the number of folds in which each feature was selected (if selected in at least one fold). Note that stage 0 refers to the warm-up stage.

Feature	Location	Direction	Stage	Number of folds
G	–	–	–	28/28
BW	–	–	–	28/28
RMS	lower back	mediolateral	1	12/28
SD	lower back	anterior-posterior	2	11/28
RMS	lower back	anterior-posterior	2	3/28
VAR	lower back	total	0	2/28
VAR^{-1}	left or right tibia	anterior-posterior	0	2/28
SD^{-1}	left or right tibia	anterior-posterior	0	1/28
AVG^{-1}	lower back	mediolateral	1	1/28
RMS	lower back	mediolateral	0	1/28
P^{-1}	left or right tibia	mediolateral	0	1/28
P	lower back	mediolateral	1	1/28
VAR^{-1}	left or right tibia	anterior-posterior	1	1/28
SD^{-1}	left or right tibia	vertical	0	1/28
