# Supplementary material for: Data fusion of body-worn accelerometers and heart rate to predict VO2max during submaximal running
Source: PLoS One. 2018 Jun 29;13(6):e0199509. doi: 10.1371/journal.pone.0199509 (PMC6025864; doi:10.1371/journal.pone.0199509)
Supplement: S3 Table — (PDF) [file pone.0199509.s003.pdf]

S3 Table. Selected features for $\mathbf{F_4}$. There were 28 folds in the leave-one-subject-out cross-validation. This table shows the number of folds in which each feature was selected (if selected in at least one fold). Note that stage 0 refers to the warm-up stage.

**Feature**	**Location**	**Direction**	**Stage**	**Number of folds**
G	–	–	–	28/28
BW	–	–	–	28/28
HR^{-1}	–	–	0	28/28
VAR^{-1}	left or right tibia	total	0	27/28
SD^{-1}	left or right tibia	total	0	1/28
